# Multiclass Classification of Agro-Ecological Zones for Arabica Coffee: An Improved Understanding of the Impacts of Climate Change

**DOI:** 10.1371/journal.pone.0140490

**Published:** 2015-10-27

**Authors:** Christian Bunn, Peter Läderach, Juan Guillermo Pérez Jimenez, Christophe Montagnon, Timothy Schilling

**Affiliations:** 1 International Center for Tropical Agriculture (CIAT), Km 17, Recta Cali-Palmira, Apartado Aéreo, 6713, Cali, Colombia; 2 International Center for Tropical Agriculture (CIAT), Hotel Seminole, 2 Cuadras al Sur, Managua, Nicaragua; 3 World Coffee Research, 578 John Kimbrough Blvd, College Station, Texas, 77843–2477, United States of America; Universidade de Vigo, SPAIN

## Abstract

Cultivation of *Coffea arabica* is highly sensitive to and has been shown to be negatively impacted by progressive climatic changes. Previous research contributed little to support forward-looking adaptation. Agro-ecological zoning is a common tool to identify homologous environments and prioritize research. We demonstrate here a pragmatic approach to describe spatial changes in agro-climatic zones suitable for coffee under current and future climates. We defined agro-ecological zones suitable to produce arabica coffee by clustering geo-referenced coffee occurrence locations based on bio-climatic variables. We used random forest classification of climate data layers to model the spatial distribution of these agro-ecological zones. We used these zones to identify spatially explicit impact scenarios and to choose locations for the long-term evaluation of adaptation measures as climate changes. We found that in zones currently classified as hot and dry, climate change will impact arabica more than those that are better suited to it. Research in these zones should therefore focus on expanding arabica's environmental limits. Zones that currently have climates better suited for arabica will migrate upwards by about 500m in elevation. In these zones the up-slope migration will be gradual, but will likely have negative ecosystem impacts. Additionally, we identified locations that with high probability will not change their climatic characteristics and are suitable to evaluate *C*. *arabica* germplasm in the face of climate change. These locations should be used to investigate long term adaptation strategies to production systems.

## Introduction

Most of the world's coffee comes from the perennial tree *Coffea arabica* [1], plantations of which are productive for 20–50 years. Climate controls where coffee can be grown [2]. Arabica coffee requires a climate with annual mean temperatures of about 20°C and over 1200 mm annual rainfall to be economically viable [2]. Temperatures over 30°C for extended periods reduce yields[3], while frost for a few days damages or even kills the plant [4]. A short dry period of less than 40mm precipitation per month increases yield and promotes uniform flowering, but more than 3 dry months reduces yield [5].

The livelihoods of about 25 million small producers globally depend on arabica coffee [6]. Because arabica requires a specific climate within narrow limits, these growers will see both yield and quality fall as the climate changes. Rising temperatures were predicted to reduce yields below economic viability in Veracruz, Mexico by 2020 [7]. For Chiapas in Mexico projections suggest that rising temperatures will eliminate coffee below 1100 masl by 2050 [8]. In the center of origin of C. *arabica*, the Ethiopian highlands, the climatic niche of the indigenous varieties may disappear by 2080 [9]. Brazil, which produces a third of the global crop of arabica coffee, is projected to lose up to 95% of the suitable area by 2100 [10]. A study of the global impact of climate change predicted that the area suitable for arabica coffee will be reduced by 50% by 2050, mostly caused by higher temperatures [11]. A more recent study showed that these impacts vary by region [12].

It is a challenge to develop unambiguous strategies to adapt to the projected changes in climate. For well researched crops like wheat, rice or maize, mechanistic simulation models are available to estimate the effects of changed climate on crop performance [13]. They can also be used to forecast how agronomic management might be adapted to changed conditions. The model available for coffee (Caf2007), however, could not be applied on larger spatial scales due to limited spatial data [14], limited knowledge of the crop’s physiology [15], and its specification for plot scale application [16].

Alternatively, the Veracruz [7] and Brazil [10] studies extrapolated known climatic limits of arabica coffee in their respective regions to forecast the distribution of future climates suitable for arabica coffee. While useful, the results could not be readily extrapolated globally. Other studies used larger data sets and methods of machine learning to predict the spatial distribution of coffee species [11,12,17]. As applied to coffee, these analyses relied on whether a site’s future climate lay within the range of those that determine current distribution. The models estimated the future distribution of arabica coffee with high confidence, but they only considered a binary distinction between suitable and unsuitable climate. This allowed the reliable identification of locations that will likely transition to other crops in the future. However, to guide adaptation research a better identification of the climatic characteristics of the impacted regions will be necessary. Furthermore, in regions that remain suitable guidance is needed to distinguish zones that will require systemic or incremental adaptation measures [18].

To address these issues, we chose an agro-ecological zoning (AEZ) approach. AEZs for arabica coffee were defined in Brazil using overlay maps of limiting climatic factors [19], or by cluster analysis of several variables in Colombia [20]. In viticulture future changes in wine growing zones have been projected using the Random Forest (RF) algorithm on local [21] and continental scale [22]. We extended this application to global scale to analyze how the different climates of each coffee AEZ will be affected by climate change. We used this analysis to suggest options to adapt and to identify homologous sites to facilitate technology transfer.

A recent review concluded that a globally-coordinated breeding program was needed to confront the negative impacts of climate change[23]. We therefore show how the AEZ approach might be used to select sites for multi-location variety trials that such a program would require.

## Data and Methods

We used WorldClim’s bioclimatic variables [24] to define AEZs suitable for arabica coffee. Although soil attributes, aspect, and local microclimate determine crop performance at local scales, they are unimportant in defining the global distribution of AEZs. We assembled a database of geo-references of sites where *C*. *arabica* is currently grown throughout the world. On the climate data for these sites we then used cluster analysis to define the AEZs that are suitable to grow arabica coffee. Next we trained the RF algorithm on the AEZ definition as response variable and the climate variables as independent variables. These RF models were extrapolated on maps of both current and future climates to predict the changes that each AEZs will confront with climate change. As a demonstration of how the method might be used, we identified possible sites for an international multi-location variety trial (IMLVT).

### Database of locations of arabica coffee

We used data of the current distribution of arabica coffee to define the climates that are suitable for cultivation. The data came from four sources:

1. Geo-referenced coffee farms from a database of the location of 100,000 farms developed by the International Center for Tropical Agriculture (CIAT) and its collaborators [11];
2. Geo-referenced municipalities in Brazil that produce arabica coffee [25]; Similarly, we produced a set of 5,666 locations for Indonesia by sampling within polygons where we knew arabica is grown and stratification based on region-specific ranges of altitude [17]
3. For those regions where neither (1) nor (2) were available, we identified coffee plantations from Google Earth images [11]; and
4. The Global Biodiversity Information Facility [26].

**Fig 1:**
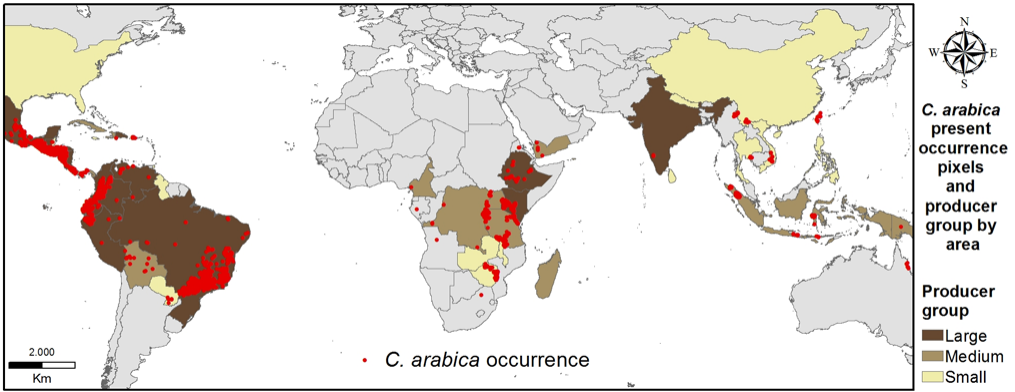
Distribution of occurrence pixels (5 arc minutes, red dots). The brown colors indicate producer groups by area of arabica coffee harvested in each country [27].

The raw database contained a total of 124,820 geo-referenced locations growing *C*. *arabica*. Because coffee farms are often small, to avoid spatial bias we reduced the database to unique pixel cells on a 5 arc-minute grid, which we call “occurrence pixels”. We stratified the database to contain only locations with elevations above 100 masl. We also removed as outliers sites for which one or more environmental variable exceeded 3.5 standard deviations from the mean. Fig 1 shows the distribution of the 3545 occurrence pixels in the final dataset together with the distribution of arabica area harvested.

### Climate data

For the current climate (1950–2000), we used the WorldClim data set at 5 arc-minute resolution [24]. WorldClim provides data of monthly precipitation, mean monthly minimum and maximum temperatures, and 19 bioclimatic variables derived from these data. We complemented the latter with another derived variable of consecutive months with less than 40mm precipitation (Table 1). As we point out in the introduction, a short dry season increases yields but dry periods longer than three months reduce yields or require irrigation.

**Table 1:** Bioclimatic variables used and mean values at occurrence pixels under current and 2050s conditions; the values for 2050s were calculated as mean impact across 19 GCMs.

Type	Bioclimatic variable	Description	Current mean	2050s mean	Unit	Clustering^1^
	BIO 1	Annual mean temperature	**20.6**	**22.5**	**°C**	
	BIO 2	Mean diurnal range (mean of monthly (max temp—min temp))	**11.6**	**11.7**	**°C**	
	BIO 3	Isothermality (BIO2/BIO7) (*100)	**72**	**71**	**-**	**X**
	BIO 4	Temperature seasonality (standard deviation *100)	**136.9**	**143.9**	**°C**	
	BIO 5	Max temperature of warmest month	**28.5**	**30.5**	**°C**	**X**
**Temperature**	BIO 6	Min temperature of coldest month	**12.2**	**13.8**	**°C**	**X**
	BIO 7	Temperature annual range (BIO5-BIO6)	**16.3**	**16.7**	**°C**	
	BIO 8	Mean temperature of wettest quarter	**21.6**	**23.4**	**°C**	
	BIO 9	Mean temperature of driest quarter	**19.3**	**21.2**	**°C**	
	BIO 10	Mean temperature of warmest quarter	**22.1**	**24.0**	**°C**	
	BIO 11	Mean temperature of coldest quarter	**18.7**	**20.4**	**°C**	
	BIO 12	Annual precipitation	**1637**	**1645**	**mm**	**X**
	BIO 13	Precipitation of wettest month	**280**	**289**	**mm**	
	BIO 14	Precipitation of driest month	**33**	**31**	**mm**	
**Precipitation**	BIO 15	Precipitation seasonality (coefficient of variation)	**66**	**67**	-	
	BIO 16	Precipitation of wettest quarter	**739**	**749**	**mm**	
	BIO 17	Precipitation of driest quarter	**122**	**121**	**mm**	
	BIO 18	Precipitation of warmest quarter	**492**	**479**	**mm**	**X**
	BIO 19	Precipitation of coldest quarter	**211**	**224**	**mm**	
	BIO 20	Number of consecutive months < 40mm precipitation	**2.5**	**2.6**	**-**	**X**

^1^ X = variables used for agglomerative clustering.

To predict climate in the period 2040 to 2069 (2050s), we used 19 global climate models (GCMs) from the *Fifth Assessment Report* (AR5) of the Intergovernmental Panel on Climate Change (IPCC) [28]. We chose the representative concentration pathway (RCP) 6.0, an intermediate scenario in which radiative forcing continues to increase until the end of the century [29].We downscaled the GCM outputs using the delta method [30], which computes the difference between model outputs for current conditions and the 2050s. We interpolated these data to 5 arc-minutes resolution and applied them to the WorldClim data for current climate and recalculated the bioclimatic variables (Table 1).

### AEZs for arabica coffee

We transformed all 20 bioclimatic variables to z-scores. For those variables that were highly correlated (Pearson coefficients |r|> 0.7), we kept the one that we judged most informative in the coffee context and discarded the others.

We then performed an agglomerative cluster analysis on the occurrence pixels using the Ward algorithm in the statistics software R [31]. We determined the final number of clusters using the indices of Ratkowsky and Lance [32] and Calinski and Harabasz [33] and by judging the dendrogram of distances between clusters. From the clusters we described AEZs classified by the bioclimatic variables that define each of them. We tested statistical significance of differences of climate data between the AEZs by one-way analysis of variance in R [31]. We based the AEZ descriptions on the differences of the group means from the grand mean and calculated their confidence intervals using R’s multcomp package [34].

### Current and future spatial distribution of AEZs for arabica coffee

We used the Random Forest package [35] to classify the climate in each pixel into AEZs for arabica coffee. We then examined the spatial distribution of each of the defined AEZs to assess the climate of global coffee growing regions, and to evaluate how climate change will affect them.

The Random Forest package creates an ensemble of decision trees and selects the mode of the individual trees, which reduces the risk of generating an overconfident classification (over fitting) [35]. We trained the algorithm with random samples of occurrence pixels within the AEZs and a random background sample of pixels within coffee-producing countries that did not have coffee. From the occurrence pixels in each AEZ group, we selected samples the same size of the smallest AEZ group and used 2.5 times as many background samples. For binary classification problems a 1:1 sampling ratio is recommended to avoid the preferential prediction of the majority class [36]. The sampling ratio we chose accounted for the trade-off between the multi-class AEZ classification, as well as the binary classification in to suitable classes and unsuitable background locations. Additionally, we constrained the background samples to contain only pixels with annual mean temperatures within the range as the occurrence pixels to exclude unfeasible locations [36]. We used all 20 bioclimatic variables as independent variables (Table 1). For each RF model we grew 1000 decision trees with seven variables selected at each node and replicated this process three times.

To make the process more robust, we divided the training sample into five random groups and trained the package five times, withholding one of the groups each time. In summary, we drew three random samples from the entire population of occurrence pixels, from each of which we drew five random subsamples to give 15 individual models trained. We extrapolated these onto maps of the 20 bioclimatic variables and determined the modal value across all 15 model results. We obtained maps of each AEZ plus class “0” background sites where Arabica coffee is unlikely to be cultivated. We repeated the process for the 2050 data. The most likely future AEZ was determined for each pixel by the mode across the results for the 19 GCMs.

To evaluate the classification we used the area under receiver operating characteristic curve (AUC), which has values 0–1. An AUC of 0.5 indicates that the performance was no better than random sampling, while 1.0 is perfect classification. We used the standard AUC to evaluate the capacity of individual models to correctly discriminate occurrence pixels from the background sample. The definition of the AUC measure can be extended to multiclass problems by averaging all pairwise AUC comparisons to a multiclass AUC [37]. We used this measure to evaluate the discrimination of AEZs by the models.

### Example: Identify potential trial sites

We classified pixels to be appropriate sites for variety evaluation in the long-term if the characteristics of the climate were stable over time and the classification was unambiguous. We therefore defined three conditions:

1. All repeats of the RF classification step had to agree on the AEZ classification under current conditions;
2. All modal classifications across the 19 GCM’s had to agree, and
3. The classifications of (1) and (2) had to be identical in both time steps.

We produced additional maps that indicated sites with less stringent classification if there was 80% agreement with the conditions above.

## Results

### AEZs for arabica coffee production

Among the temperature factors the annual mean temperature and the annual total precipitation are most frequently mentioned in the literature to describe the spatial distribution of climatic suitability for coffee production (e.g. [5]). Furthermore, maximum and minimum temperatures were shown to be influential, but also temperature variability [15].

At the 3545 occurrence pixels of *C*. *arabica* Bio5 (the mean maximum temperature of the warmest month) and Bio6 (the mean minimum temperature of the coldest month) were correlated with Pearson coefficients |r| = 0.42. This was below the threshold (|r|> 0.7), so we included them for clustering. In contrast, Bio1 (the annual mean temperature) correlated with several other temperature variables so we excluded all of them. Of the variables that represent temperature variability, Bio3 correlated least with Bio5 and Bio6. It is the ratio of the mean monthly temperature range to the annual range (Bio2/Bio7*100) (|r|≈ 0.4), and we included it in the analysis (Table 1).

The literature identifies annual precipitation (Bio12) and the length of the dry season (Bio20) as the most important precipitation-related variables that influence the yield of coffee. Their Pearson coefficient was |r| = 0.56 so we included them in the analysis. In general, coefficients among precipitation variables were high and we excluded them. The exception was variable Bio18, the precipitation of the warmest quarter, which had acceptable coefficients with both Bio12 (|r_BIO12_| = 0.45) and Bio20 (|r_BIO20_| = 0.21) and low coefficients with most other precipitation variables; we therefore included it in the analysis (Table 1).

**Fig 2:**
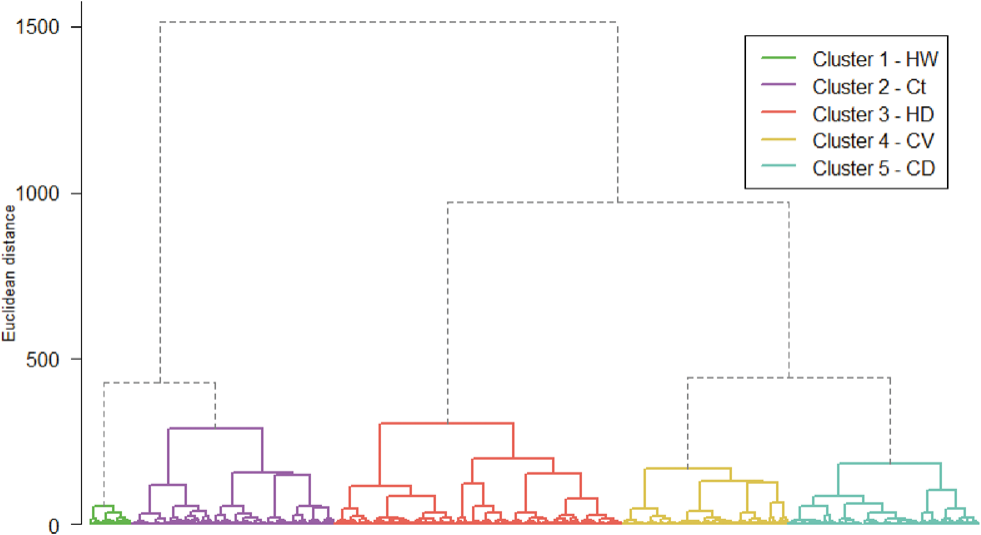
Dendrogram of agglomerative clustering. On the vertical axis is Euclidean distance. The final definition of clusters is indicated by the color codes: Green-“Hot-Wet”, Violet-“Constant”, Red-“Hot-Dry”, Yellow-“Cool-Variable”, Turquoise–“Cool-Dry”.

We obtained five distinct agro-ecological zones (AEZs, Fig 2) for arabica coffee within the 3545 occurrence pixels based on six standardized bioclimatic variables. We described the AEZs in terms of their climatic characteristics. For some better insight into of Bio3 we included the related variables Bio2 (Mean diurnal range) and Bio7 (Annual temperature range).

**Fig 3:**
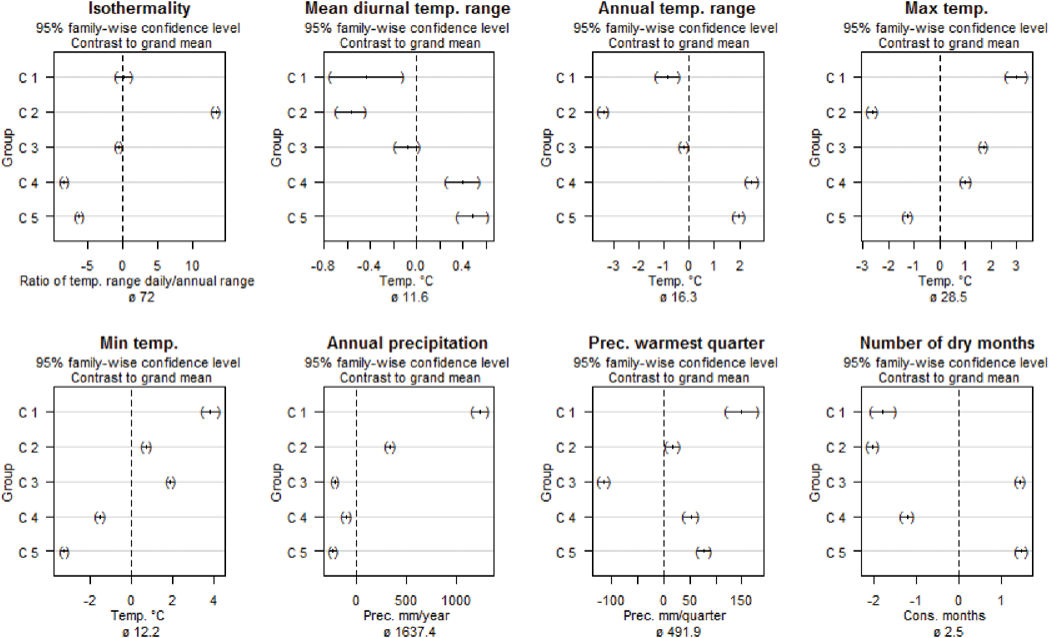
Confidence intervals of group compared with the grand mean for descriptor variables. Group labels are the cluster numbers (C 1–5) in the dendrogram (Fig 2). The dashed line is the grand mean, the value for which is given at the bottom.

One way analysis of variance showed highly significant differences between all AEZs for all variables (at p < 0.001). We summarized the five AEZs as follows (the list numbers correspond to the groups in Fig 2):

1. “Hot-wet” (HW), characterized high maximum temperature in the warmest month, high annual precipitation, a short dry season and a humid warmest quarter of the year (Fig 3).
2. “Constant” (Ct), characterized by lacking temperature seasonality. It had the highest isothermality, and the lowest mean values for daily and annual temperature range. Ct’s precipitation is similar to the HW zone with high annual precipitation and a short dry season (Fig 3).
3. “Hot-dry” (HD), characterized by high maximum temperatures were high and no cold month. The annual total precipitation is low and has the lowest precipitation in the warmest quarter of all groups and a long dry season (Fig 3).

The last two groups of occurrences are characterized by low minimum temperatures.

5. “Cool-variable” (CV) characterized by the highest annual temperature range, and the lowest mean isothermality. Precipitation is moderate.
6. “Cool-dry” (CD), characterized by the lowest minimum temperature of the coldest month but also the lowest annual precipitation with a long dry season.

### Current and future spatial distribution of clusters

**Fig 4:**
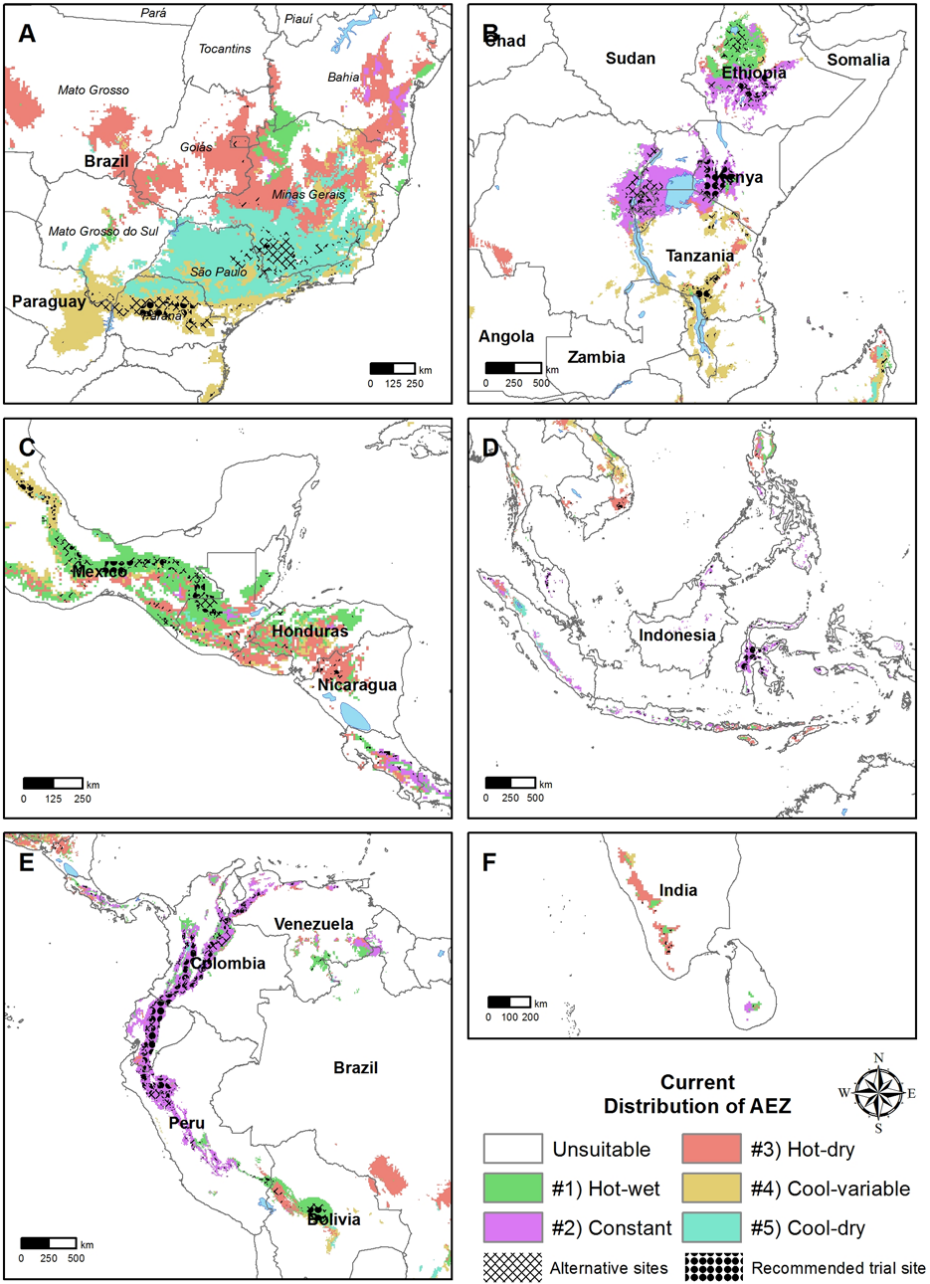
Distribution of AEZs in regions important for arabica coffee; A Brazil; B East Africa; C Central America; D Indonesia; E Colombia; F India; Colored grid cells represent the agro-ecological zone; dots indicate sites recommended for trial sites, hatching alternative locations with less model agreement.

**Fig 5:**
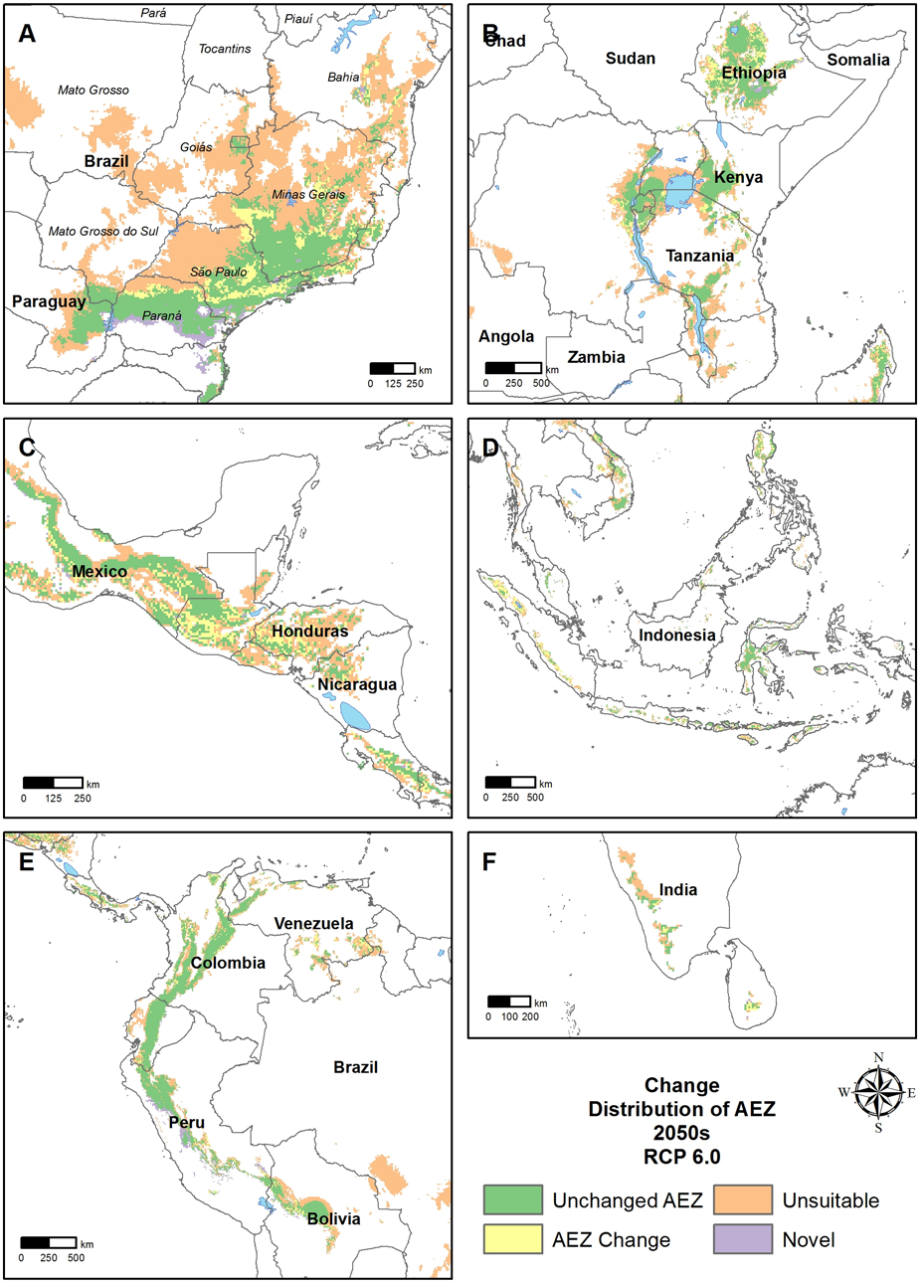
Change of agro-ecological zone in important arabica coffee production regions until the 2050s; A Brazil; B East Africa; C Central America; D Indonesia; E Colombia; F India

The RF classification gave maps of current distribution of the AEZs (Fig 4) and the changes that climate change will bring as forecast by the 19 GCMs (Fig 5).

The Ct AEZ is mostly in highlands close to the equator: Colombia, Ethiopia, Kivu lake region in Central Africa, Kenya and Indonesia (Fig 4B, 4D and 4E). Regions towards the latitudinal margins were characterized by more variability. Southern Brazil was mostly dominated by the related AEZs HD, CV and CD. The latter two AEZs show a high seasonal temperature variation and low minimum temperatures. While in southern Brazil the CV AEZ has a long dry season, CD AEZ has lower maximum temperatures and a shorter dry season. In central Brazil, the HD AEZ region has high maximum temperatures and a strong dry season (Fig 4A). In the Central America region all AEZs were represented but HW and HD with high maximum temperatures are most prevalent. Some regions experience high rainfall, however, while others are characterized by lower precipitation and longer dry seasons. Southern Mexico was typical for the HW AEZ with a hot and wet climate. Nicaragua was representative of the HD AEZ with a long dry season and high temperatures. Costa Rica is an exception in this region with most of its coffee region being of Ct climate with stable and moist conditions (Fig 4C).

By 2050, the spatial distribution of AEZs changes little, with Ct still mostly around the equator. The extent of the distribution will be reduced, however, especially in Brazil’s coffee growing regions, with most of the HD AEZ pixels becoming unsuitable. The CD AEZ will also be reduced substantially, especially in western São Paulo state. The areas that are currently in the CV AEZ zone largely remain of the same climate. There will not be any migration to the south (Fig 5A). Changes in Central America will be similar to those in Brazil with most of the HD AEZ mostly becoming unsuitable. The location of the other AEZs persists but reduced extent in extent (Fig 5C).

Under current conditions, arabica coffee grows on 7.2% of the total pixels in the latitudinal belt 30°N– 33°S. The Ct AEZ accounts for 26% of all suitable pixels, which is a little more than the HD AEZ with 25%. The other three AEZs shared the remainder 49%. The total share of suitable pixels in the 30°N– 33°S belt will be halved to 3.6% by the 2050s. The Ct AEZ will be less affected by climate change than the others and will make up for 34% of the area that remains suitable for arabica coffee. Most of the loss will be in the HD AEZ, while the other three AEZs will be reduced proportionally so that their relative share changes little (Table 2).

**Table 2:** Distribution of grid cells in the agro-ecological zones under current and 2050s conditions.

Climate		Unit	Hot-wet	Constant	Hot-dry	Cool-variable	Cool-dry	Total
**Current**	Pixel count	-	8943	14869	14337	11637	6479	56265
	Pixel share	%	16	26	25	21	12	7.2^1^
	Median elevation	masl	946	1578	807	825	704	1024
**2050**	Pixel count	-	4992	9710	4248	6944	2859	28753
	Pixel share	%	17	34	15	24	10	3.6^1^
	Median elevation	masl	1429	1954	1185	812	835	1362

^1^ Percent share of all grid cells within a latitudinal belt 30°N– 33°S

The median elevation of all suitable pixels was 1024 masl but the median elevations differed between AEZs. The Ct AEZ lay at 1575 masl compared with the CD AEZ at 700 masl. By 2050, the median elevation of pixels that remain suitable for arabica coffee will be more than 300m higher. The effect will differ by AEZ; the elevation of the CV AEZ will not change, while the HW AEZ will be nearly 500m higher (Table 2).

**Fig 6:**
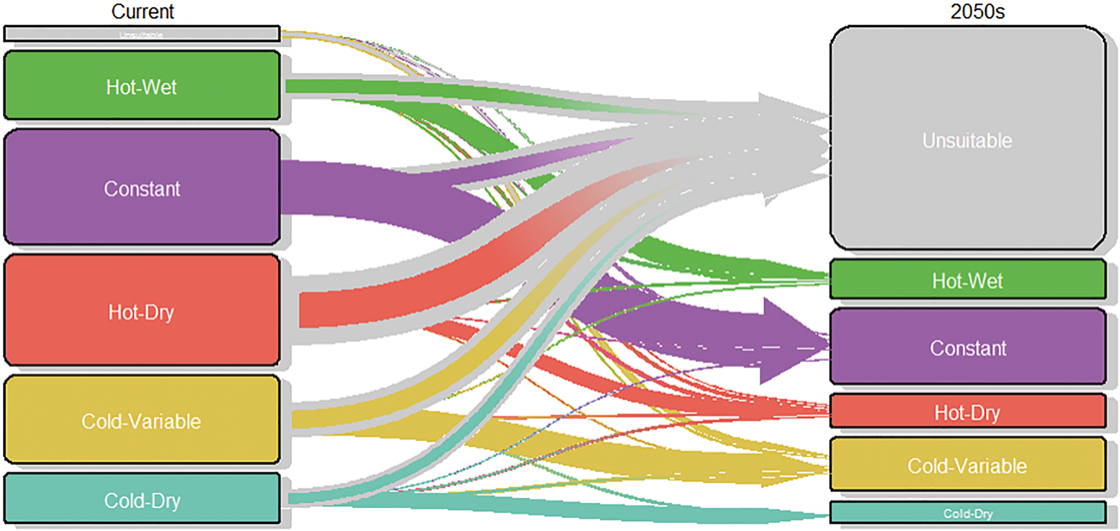
Transition plot of the fate of suitable pixels in coffee AEZs from current conditions to 2050s; size of boxes and width of transition arrows is representative of the number of pixels; the size of the box of the “unsuitable” category includes only pixels that become suitable by 2050 or were suitable with current climate.

We disaggregated the AEZs for now and 2050 to determine in which AEZ each pixel will be classified in 2050 (Fig 6). The Ct AEZ was least affected with 59% of the pixels unchanged, 4% becoming each HW and HD, and 34% becoming unsuitable. The HD AEZ will be most affected, with only 16% of current pixels remaining suitable by 2050s and 78% becoming unsuitable. Of the other three AEZs about 40% of current grid cells remain suitable and 46–49% will become unsuitable. The remainder shifted to the other AEZs (Fig 6).

Only half of the pixels that will be in the HD AEZ in 2050 currently belong to this AEZ, with the remainder coming from other zones. In contrast, nearly all future area the Ct AEZ currently belong to it. Only a small part of the area that will be suitable for arabica coffee in 2050 will be land that was previously unsuitable. Half of this novel area will be in the CV AEZ (Fig 6), such as in the southern margin of Brazilian coffee zones (Fig 4A).

The average ability of the RF algorithm to discriminate AEZs was satisfactorily high across all individual models, with the multiclass AUC averaging 0.84, which is much better than chance. The conventional AUC measure averaged 0.91, which demonstrates the robustness of the algorithm to discriminate between suitable and unsuitable pixel cells.

### Example application: Spatial distribution of robust sites

We overlaid the maps in Fig 4 with pixels that met the 80% and 100% stability criteria defined above in the Data and methods section.

We used these to make a list of recommended sites for trials for each country that is an important arabica producer. For each site within an AEZ we listed geographic location, administrative description, altitude and values of bioclimatic variables were given (Table 3).

**Table 3:** List of recommended trial sites for Nicaragua.

ID	AEZ	Lon	Lat	Country	State	District	Elev	bio_2	bio_3	bio_5	bio_6	bio_7	bio_12	bio_18	bio_20
		°	°	-	-	-	masl	0.1°C	-	0.1°C	0.1°C	0.1°C	mm	mm	months
12	Hot-dry	-85.958	13.125	Nicaragua	Jinotega	Jinotega	1330	98	70	252	113	139	1797	398	3
13	Hot-dry	-86.458	13.208	Nicaragua	Estelí	Estelí	1164	105	73	274	132	142	1669	638	5
15	Hot-dry	-86.125	13.292	Nicaragua	Jinotega	San Sebastián de Yalí	1369	101	70	262	119	143	1738	393	3
16	Hot-dry	-86.042	13.292	Nicaragua	Jinotega	San Rafael del Norte	1032	105	73	274	131	143	1486	372	3
17	Hot-dry	-86.625	13.375	Nicaragua	Madriz	San Lucas	1131	106	74	278	135	143	1929	777	3
19	Hot-dry	-86.542	13.708	Nicaragua	Nueva Segovia	Macuelizo	1290	103	70	276	130	146	1864	558	2
20	Hot-dry	-86.542	13.792	Nicaragua	Nueva Segovia	Dipilto	1370	101	69	271	126	145	1864	546	2

We could not identify robust sites for all countries that are important arabica producers. Nor could we identify potential trial sites for all the AEZs that occur in each country, for example in Nicaragua the only robust sites were in the HD AEZ. Although a part of Nicaragua’s coffee is produced in the HW AEZ, none of these pixels were classified as robust.

## Discussion

The agro-ecological zoning model developed here adds to our understanding of the climate change impacts on the production of arabica coffee globally. The global AEZ approach we used goes beyond previous regional AEZ research by demonstrating how different AEZs will be affected differently, which can be used to guide research into adaptation to climate change. The global impacts we project here agree with previous studies on the magnitude of impacts, which is a reduction of area suitable for coffee production by about 50% until the 2050s [9,11,12].

Most areas that will become unsuitable to grow arabica coffee in the future now have climates with high maximum temperatures and long dry seasons (AEZ HD). These include some areas that currently give high yields of arabica coffee (northern Minas Gerais state in Brazil, parts of India, and Nicaragua). In contrast, substantial areas that currently lie in other AEZs will become HD in the future. These scenarios will offer both challenges to and opportunities for the coffee sector. On the one hand, important coffee-producing areas will struggle to remain productive while currently less-favored areas may become more productive. Research to adapt coffee production to climate change will thus have to make arabica coffee better adapted to heat and drought stress. Other regions may have to change their agronomic practices to remain competitive, for example by learning from farmers who are currently productive in the HD AEZ.

The constant (Ct) AEZ, which has neither high nor cold temperatures, will be least affected by climate change. It occurs close to the equator in Colombia, Ethiopia, Kenya and Indonesia and produces high quality coffee. Despite the comparatively small effects of climate change on this AEZ, challenges arise due to decreased coffee quality [38]. Climate change will bring few novel pixels in the Ct AEZ so that its increase in median elevation was caused by it losing low-elevation pixels. Due to the conical form of land with elevation, there is more agricultural area at lower elevations than there is upslope. The actual area lost to production may therefore be larger than the number of pixels lost suggests. Moreover, land at high elevations is often difficult to access, is too steep for cultivation, or has shallow soil so that upward migration may not always be an option. Furthermore, land at higher elevations often has high ecosystem value or is used for forestry, which could be further barriers to upward migration of coffee.

The Hot-wet (HW) AEZ has high precipitation similar to the Ct AEZ. Temperatures are higher, but seasonal variation is average. Typical locations are southern Mexico and central Ethiopia. The HW AEZ occurs associated with both Ct and HD AEZs, sharing high maximum temperatures with the latter. The strong effect of climate change on elevation in the HW and Ct AEZs suggests that higher temperatures will be the most limiting factor. High temperatures induce dehiscence of flowers and fruits [4] and make attacks from pests like the coffee berry borer [39] more likely.

About half of the novel area for arabica coffee will be dispersed among existing coffee regions at higher elevations. Substantial areas will become suitable south of the southern margin of the Brazilian coffee region. These areas have short dry seasons but temperature variability and especially low minimum temperatures currently make them unsuitable for coffee. The higher temperatures that climate change will bring will reduce frost risk [19]. Nevertheless, there will be little expansion beyond the present latitudinal limits because temperature variability will remain a limitation.

We aimed to identify homologous climatic zones in current and future conditions, which is the basis of the AEZs. Locations that become another AEZ in the future may adapt to it guided by how growers that are currently in that AEZ manage their crops. The AEZs that we defined, however, are based on current climates, and we use the same criteria to classify future climates. But climates that we consider unusual or marginal for arabica coffee today may become more common by 2050. For example, the AEZ that dominates southern Brazil, CV, has low minimum temperatures and high temperature variability. With climate change bringing reduced frost risk, we speculate that in the future a novel AEZ with high temperature variability and high maximum temperatures could become important.

We defined the AEZs using a database of occurrence pixels where *C*. *arabica* is grown that included data from all the world’s coffee-producing regions. We excluded as outliers locations with unusual climates. It might be useful to include marginal locations, however, as the environmental limits for arabica coffee may provide insights into possible adaptation strategies. The addition of occurrence records from marginal locations such as Zambia or Yemen may improve our ability to differentiate between marginal and unsuitable climates. This could contribute to our ability to adapt to more extreme climates in the future.

Machine learning approaches, like the RF algorithm used here, have been criticized to be prone to overfit to specific variable states. We applied the algorithm carefully, choosing variables that with low levels of correlation and achieved a high classification accuracy as shown by the AUC metric. Moreover, the overall projected impact of climate change is similar to that projected in other studies [11]. On the other hand, climate change impacts on coffee will potentially be more severe as was demonstrated by models with more pessimistic emission scenario choices or when considering a longer time horizon [9,10]).

We specified that robust sites for the long-term variety trials must unambiguously represent an AEZ and that the fundamental climate characteristics will be unaffected by climatic change. We specified these conditions because variety improvement in coffee can take several decades. Fundamental changes in the climate during the course of a long-term trial would invalidate comparison of data gathered over many years. For the identification of robust sites for each AEZ we took account of variation between GCMs and selected only those sites that could be classified unambiguously. Only a small number of such pixels could be identified.

In conclusion, we therefore urge coffee research to consider climatic change carefully when taking decisions with a long time horizon such as selecting sites for variety trials. When comparing data from previous experiments, analysis often considers only the environmental parameters of interest. This approach may result in erroneous conclusions if fundamental characteristics of the climate change over the time interval under consideration.

We urge that tests of strategies to improve varieties and other agronomic measures consider the locations we identify here. Nevertheless, future research will also have to expand the environmental limits of arabica coffee to novel or marginal climates to minimize the worst impacts of climate change on the coffee sector.
